# Selective Thermal and Photocatalytic Decomposition of Aqueous Hydrazine to Produce H_2_ over Ag-Modified TiO_2_ Nanomaterial

**DOI:** 10.3390/nano13142076

**Published:** 2023-07-15

**Authors:** Shaeel Ahmed Althabaiti, Zaheer Khan, Katabathini Narasimharao, Salem Mohamed Bawaked, Soad Zahir Al-Sheheri, Mohamed Mokhtar, Maqsood Ahmad Malik

**Affiliations:** 1 Chemistry Department, Faculty of Science, King Abdulaziz University, P.O. Box 80203, Jeddah 21589, Saudi Arabiankatabathini@kau.edu.sa (K.N.);; 2 Department of Chemistry, Faculty of Natural Sciences, Jamia Millia Islamia, New Delhi-110025, India

**Keywords:** H_2_ production, Ag-modified TiO_2_, photocatalysis, hydrazine decomposition

## Abstract

An Ag-modified TiO_2_ nanomaterial was prepared by a one-pot synthesis method using tetra butyl titanate, silver nitrate, and sodium hydroxide in water at 473 K for 3 h. X-ray diffraction, scanning electron microscopy, and transmission electron microscopy were used to determine the structure and morphology of the synthesized Ag-modified TiO_2_ nanomaterial. The diffuse reflectance UV-visible and photoluminescence spectroscopy results revealed that metallic Ag nanoparticles decreased the optical band gap and photoluminescence intensity of the TiO_2_. In addition, the Raman peak intensity and absorbance were increased after Ag modification onto TiO_2_. The photocatalytic efficiency of the synthesized samples was tested for decomposition of aqueous hydrazine solution under visible light irradiation. The photocatalytic efficiency of Ag-modified TiO_2_ nanomaterials was higher than that of bare TiO_2_ and Ag metal NPs due to the synergistic effect between the Ag metal and TiO_2_ structures. In addition, the surface plasmon resonance (SPR) electron transfer from Ag metal particles to the conduction band of TiO_2_ is responsible for superior activity of TiO_2_-Ag catalyst. The Ag-modified TiO_2_ nanomaterials offered a 100% H_2_ selectivity within 30 min of reaction time and an apparent rate constant of 0.018 min^−1^ with an activation energy of 34.4 kJ/mol under visible light radiation.

## 1. Introduction

Hydrogen has been considered as an alternate source of clean energy. The production of hydrogen from the splitting of water [1,2], photocatalysis [3,4], and decomposition of hydrogen chemical storage materials with different catalysts [5,6], especially nanosized oxides, have been the subject of interest for many years [7]. Hydrazine hydrate (N_2_H_4_·H_2_O) is a well-known hydrogen storage material with an excellent hydrogen storage content (8.0 wt.%), and it produces H_2_ and N_2_ when it is completely decomposed, which has been considered as a green process [8]. Hydrazine hydrate can decompose via two major reaction pathways: (i) decomposition to H_2_ and N_2_ (Equation (1), complete decomposition) and (ii) decomposition to N_2_ and NH_3_ (Equation (2), incomplete decomposition) [9]. Complete decomposition is the desired process to produce H_2_ with high yields without the formation of byproducts such as NH_3_, which could lead to catalyst deactivation and environmental pollution. However, the formation of NH_3_ is thermodynamically favorable; therefore, utilization of a suitable catalyst that selectively decomposes hydrazine hydrate to H_2_ and N_2_ is necessary [9].

(1) $$NH_{2}-NH_{2}\to 2H_{2}+N_{2}\;\left(Complete\;decomposition\right)\;$$

(2) $$3NH_{2}-NH_{2}\to 4NH_{3}+N_{2}\;\left(Incomplete\;decomposition\right)\;$$

Different types of catalysts such as Rh–Ni/GO, Ir-Ni stabilized by CTAB, Fe-B/MWCNTs, and TiO_2_-modified Rh (I) coordinated catechol phosphane ligand were used for the selective decomposition of hydrazine hydrate to produce H_2_. However, these catalysts are expensive catalytic systems, which require multiple synthetic procedures [10]. Semiconductor-based photocatalysts such as TiO_2_, ZnO, and g-C_3_N_4_, etc., are widely used for solar-light-driven photocatalytic degradations of pollutants, the oxidation of VOCs, CO_2_ reduction, and hydrogen generation [11]. Nowadays, TiO_2_-based photocatalysts receive great interest due to their photocatalytic activities, chemical stabilities in aqueous solutions, and favorable band gap energies [12,13]. However, its broad band gap (3.0 eV for rutile and 3.2 eV for anatase) and high e^−^/h^+^ recombination restrict its photocatalytic performance, resulting in confined use [14]. Moreover, pure TiO_2_ has poor photoelectronic transition efficiency because it can only absorb UV light (λ < 350 nm), which accounts for around 4% of the solar spectrum, into photocatalytic processes [15,16]. On the other hand, metal-modified TiO_2_ has been used as a photocatalyst under visible and solar light irradiations for H_2_ generation via water splitting [17]. Therefore, various efforts have been devoted to tuning the band gap and electronic structure of TiO_2_ by doping transition metals [18,19], nonmetals [20], noble metals [21], and through sensitization with dyes [22] for the improvement of its photocatalytic activity in the visible light region. It is well established that the photocatalytic activity of TiO_2_ depends on the nature of metals and/or nonmetals and is extensively used for H_2_ generation from splitting reactions under visible light irradiation (λ > 400 nm) for fuel cells [23,24]. Certain noble metals, such as Pt, Au, Pd, and Ag, can be deposited on TiO_2_ to increase the H_2_ production rate by photocatalysis [25]. A UV-active TiO_2_ photocatalyst can be successfully modified to a visible-light-induced photocatalyst by modifying the surface of the TiO_2_ by the successful deposition of metal ions [26,27]. It is beneficial to develop a TiO_2_-based photocatalyst for H_2_ generation, involving hydrazine decomposition under visible light radiation. Kumar et al. [28] and Luo et al. [29] used Rh-doped TiO_2_ and face-centered indium–ionic liquid nanocubes as the catalysts for H_2_ production by hydrazine decomposition under visible light irradiation. However, there is no report available in the literature on photocatalytic H_2_ production by hydrazine decomposition with Ag-modified TiO_2_ nanomaterials under visible light. Hence, this research aims to demonstrate the utilization of an Ag-modified TiO_2_ nanomaterial for the photocatalytic decomposition of hydrazine for H_2_ generation.

In this contribution, an Ag-modified TiO_2_ nanomaterial was synthesized by following the one-pot synthesis approach and used for the photocatalytic decomposition of hydrazine under visible light. The H_2_ generation rates were studied by varying the hydrazine concentrations, amounts of photocatalyst, pHs, and reaction temperatures. However, fast H_2_ generation within a short reaction time has yet to be examined. Furthermore, the synthesized materials were characterized with different techniques to investigate their physicochemical properties and relationships to photocatalytic performance.

## 2. Experimental Procedure

### 2.1. Chemicals

The metal precursors titanium (IV) butoxide (Ti(O-CH_2_-CH_2_-CH_2_-CH_3_)_4_, molar mass = 340.3 g/mol, reagent grade, 97%) and silver nitrate (AgNO_3_, 99%) were received from Sigma Aldrich and used for the synthesis of the Ag-modified TiO_2_ nanomaterial. Hydrazine anhydrous (NH_2_NH_2_, 98%) and sodium hydroxide (NaOH) were utilized as the sources of hydrogen and to control the pH of the solution, respectively. Double-distilled water was used for the preparation of all solutions. The NaOH solution was prepared and standardized with standard oxalic acid using phenolphthalein as an indicator.

### 2.2. Synthesis of Ag-Modified TiO_2_ Nanomaterial

The one-pot synthesis method was employed to prepare Ag-modified TiO_2_ nanomaterials with slight modifications [30]. In a typical procedure, a solution containing titanium (IV) butoxide (0.05 mol/L of 5.5 mL) and silver nitrate (20 mL of 1.0 mM) was placed in a round bottom flask, and pure nitrogen gas was bubbled through the solution to maintain an inert atmosphere. The pH of the contents was adjusted to ca. 9.5 by adding a diluted NaOH solution, promoting the formation of Ti and Ag hydroxides. The obtained suspension was then heated at 473 K for 3 h. After these steps, the solid material was recovered by centrifugation and washed with acetone and water. The obtained material was dried under vacuum for 12 h at 353 K and stored in a brown glass container. The bare TiO_2_ nanoparticles sample was obtained without the addition of silver nitrate solution (Equations (3) and (4)).

(3) $$Ti{\left(O-{\left(CH_{2}\right)}_{3}-CH_{3}\right)}_{4}+4NaOH\to Ti{\left(OH\right)}_{4}+4CH_{3}-{\left(CH_{2}\right)}_{3}ONa\;$$

(4) $$Ti{\left(OH\right)}_{4}+Heat\to TiO_{2}\;$$

### 2.3. Photocatalytic Activity Test

The photocatalytic activity of the synthesized pure TiO_2_ and Ag-modified TiO_2_ nanomaterial for the decomposition of aqueous hydrazine solution in the absence and presence of visible light was tested using a laboratory-built reactor setup. In a typical experiment, the Ag-modified TiO_2_ nanomaterial (20 mg) was added to 10 mL of deionized water in a reaction vessel; the required hydrazine solution was then injected into the reaction mixture, and then the reaction vessel was purged with an inert gas (He) flow of 90 mL/min for 30 min to remove the air. Finally, the total contents were subjected to monochromatic light irradiation (red LED lamp) with a maximal wavelength (λ_max_) of 600 nm and a power of 25 mWcm^−2^. For qualitative measurements for hydrogen gas, a Honeywell XCD Sense point gas detector was used. The gas detector was mounted on a mounting plate attached to the surface of a frame. The outlet of the reactor was connected to the sensor gas cap using tubing. When the produced gas reached the detector, the screen displayed the H_2_ concentration (vol% in the gas stream) during the run (approximately 3 min to obtain a reading). The volume of generated gases (hydrogen + nitrogen) was determined using the water displacement method at room temperature under pseudo-first-order conditions (excess of hydrazine) [31,32]. The rate constants (*k*_obs_) were calculated with Equation (5).

(5) $$k_{obs}=\frac{1}{t}ln\left(\frac{V_{\alpha }-V_{0}}{V_{\alpha }-V_{t}}\right)\;$$

*V*_α_ = final volume of generated H_2_ + N_2_ at the completion of the reaction, *V*_0_ = volume of generated H_2_ + N_2_ at *t* = 0 min, and *V*_t_ = volume of generated H_2_ + N_2_ at different time intervals [33]. The *k*_obs_ were estimated from the slopes of *ln*(*V*_α_ − *V*_0_/*V*_α_ − *V*_t_) plots versus times at different concentrations of hydrazine, amount of catalyst, and temperatures ranging from 295 K to 323 K. The gases released during the reaction were passed through a solution of Nessler’s reagent to ensure the formation of ammonia as the other side product. The same kinetic experiments were also performed in the dark. The catalyst reusability experiments were conducted over the recovered catalyst by adding the same amount of hydrazine to the reaction vessel after the complete evolution of hydrogen. A total of five cycles were tested using synthesized catalysts.

### 2.4. Characterization of Synthesized Nanomaterials

An X-ray diffractometer (RXD, Ragaku D-max 2200) was employed to establish the crystalline nature of the catalyst. Scherer’s equation: d (hkl) = 0.94 λ/β(cos2θ), where d (hkl) was the average crystallite size (nm), λ was the source of CuKα radiation applied (0.154 nm), and β was the full width at half maximum intensity of the XRD reflection observed at 2θ = 25.2° was used to determine the crystallite size. A transmission electron microscope (TEM, JEOL, JEM-1400Flash) and a scanning electron microscope (JEOL) were used to determine particles’ morphologies, shapes, sizes, and distributions in the synthesized catalysts. To measure TEM/SEM images, a small amount of catalyst was dispersed in ethanol and deposited on the carbon-coated copper grid. The solvent evaporated at room temperature, and then the images were recorded using an operating power of 120 kV. Energy-dispersive X-ray spectroscopy (EDS) determined the elemental composition of samples. Diffuse reflectance UV-visible spectra were recorded using a UV2550 spectrophotometer (Shimadzu, Japan). Photoluminescence spectra (PLS) were measured using an F-7000 fluorescence spectrophotometer (Hitachi, Japan). The textural properties, such as the BET surface areas, pore volumes, and pore sizes, of the catalysts were determined by nitrogen physisorption measurements using an Autosorb AsiQ (Quantachrome, USA) surface area analyzer. A SPECS GmbH X-ray photoelectron spectrometer measured the X-ray photoelectron spectra (XPS). A standard dual anode excitation source with Mg Kα (1253.6 eV) radiation was used at 13 kV and 100 W.

## 3. Results and Discussion

### 3.1. Catalysts Characterization

**Figure 1:**
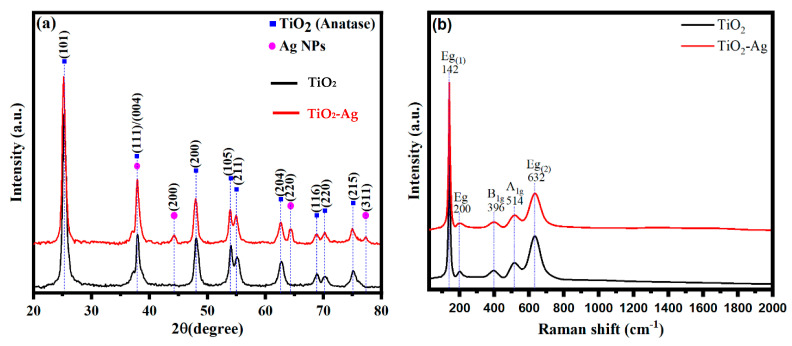
(**a**) XRD of TiO_2_ and Ag-doped TiO_2_ and (**b**) Raman spectra of TiO_2_ and Ag-deposited TiO_2_.

The crystalline phases present in the synthesized TiO_2_ nanomaterials were analyzed by recording the XRD patterns of the samples. Figure 1a shows XRD patterns of the bare TiO_2_ and Ag-modified TiO_2_ nanomaterials. For the bare TiO_2_ sample, the most intense peak was observed at 2θ = 25.2°, corresponding to the (101) plane of the anatase TiO_2_ phase [34]. 

The XRD patterns were compared with JCPDS file no. PDF 021-1272 data files. The prominent diffraction peaks that appeared at 2θ = 25.2°, 37.5°, 48.3°, 52.2°, 54.4°, 62.4°, 68.9°, 70.1°, and 75.5° could have been ascribed to the (101), (111), (200), (105), (211), (204), (116), (220), and (215) lattice planes of the anatase phase of the TiO_2_, respectively [32]. The additional XRD peaks that appeared at 2θ = 37.5°, 44.3°, 64.3°, and 78.2° for (004), (200), (220), and (311) were due to the face-centered cubic metallic silver phase (JCPDS no. 04-0783). No other diffraction peaks were observed in the XRD patterns, indicating the successful synthesis of the desired crystalline phases. The peak at 2θ = 37.5° was for both the (004) and (111) planes of the metallic silver (Ag^0^) and TiO_2_ phases, respectively. Liu et al. deposited the Ag^0^ on the TiO_2_ nanosheets by a complex microwave chemical reduction method [35] and reported a similar observation. The average crystallite sizes (d) of TiO_2_ and Ag^0^ in the Ag-modified TiO_2_ nanomaterials were calculated from Debye–Scherer’s equation and were about 15 nm and 10 nm, respectively.

The plasmonic Ag nanomaterials on the TiO_2_ surface could enhance the optical properties of TiO_2_ and be used as a Raman scattering agent for detecting bioorganic molecules [36]. Therefore, the Raman spectra of the TiO_2_ and Ag-modified TiO_2_ samples were recorded. Figure 1b shows the Raman spectra of bare TiO_2_ and Ag-modified TiO_2_ samples. The prominent characteristic Raman bands were observed at 142 cm^−1^, 200 cm^−1^, 396 cm^−1^, 514 cm^−1^, and 632 cm^−1^, suggesting a pure anatase form (Figure 1b, black line). The Raman band intensity was increased due to presence of plasmonic Ag^0^ nanoparticles (Figure 1b, red line). Mills et al. [37] and Yang et al. [38] also reported a similar observation, detailing that the Raman signals’ Ag-TiO_2_ frequency increased with the amount of metallic silver in the samples. Interestingly, the band positions of TiO_2_ remained unchanged after the Ag^0^ modification in the Raman spectrum, indicating that the Ag^0^ particles dispersed on the surface and were not incorporated in the TiO_2_‘s crystal structure. The surface-enhanced Raman scattering of Ag-modified TiO_2_ could have been attributed to the surface Plasmon resonance of metallic silver nanoparticles.

**Figure 2:**
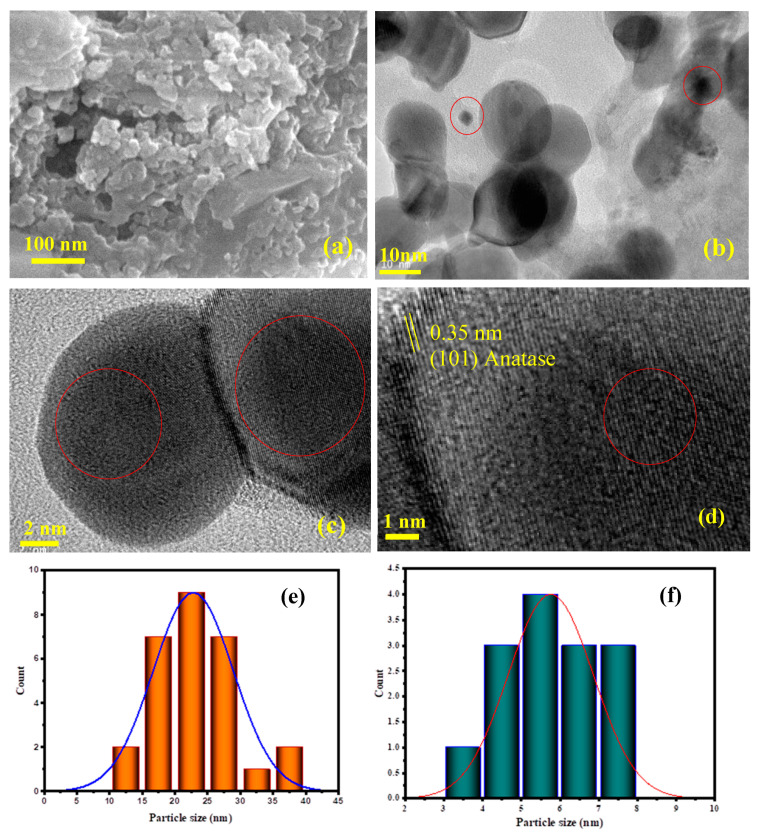
SEM (**a**) and TEM (**b**–**d**) images of Ag-modified TiO_2_ nanomaterial, (**e**,**f**) histograms for TiO_2_ (23 nm) and Ag^0^ (7 nm) nanoparticles.

The SEM and TEM images of Ag-modified TiO_2_ are shown in Figure 2a–d, respectively. The TEM images indicated that the Ag^0^ nanoparticles (black spots) were deposited onto the surface of TiO_2_ particles in an unsymmetrical manner, which led to the formation of a long aggregated spherical structure in the Ag-TiO_2_ composite structure. Yu et al. reported the synthesis of spherical TiO_2_ nanoparticles and observed a similar observation [39]. The high-resolution TEM images showed two TiO_2_ spherical particles agglomerated with each other, along with Ag-TiO_2_ interactive species on the surface. It is also clear that the high-resolution TEM showed the lattice fringes with an interplanar distance of 0.35 nm corresponding to the (101) plane of the Anatase crystal structure along the presence of dark spots corresponding to Ag metallic species. These observations suggested that the spherical Ag nanoparticles were highly dispersed onto the spherical TiO_2_ anatase nanoparticles [40]. This close interaction between Ag^0^ and TiO_2_ was believed to enhance the photogenerated electron transfer in the Ag-TiO_2_ nanomaterial [41]. The histograms obtained for TiO_2_ and Ag^0^ nanoparticles were obtained from the TEM image of the TiO_2_-Ag sample, and the results indicated the presence of TiO_2_ nanoparticles with an average particle size of 23 nm. In comparison, the sample possessed Ag^0^ nanoparticles with an average particle size of 7 nm. These observations were in accordance with the crystallite size measured from XRD results. The aqueous solution contained Ag^+^, Ti^4+^, [C_4_H_9_O]^−^, and [NO_3_]^−^ ions before precipitation by NaOH. In the solution state, Ag^+^ ions could start to receive free electrons in the solution to form an Ag^0^ metal atom (nucleation process). In the drying process, water began to evaporate. During calcination with N_2_ gas flow at temperatures from 673 K, the [C_4_H_9_O]^−^ and [NO_3_]^−^ species could decompose into some gases such as CO, CO_2_, H_2_, and NO_2_. The Ag nanoparticles started to grow after drying. The smaller Ag nanoparticles agglomerated into larger particles after calcination. It was previously reported that the calcination process under N_2_ gas flow could remove oxygen from possible Ag–O nanoparticles to produce the Ag metal nanoparticles [42].

**Figure 3:**
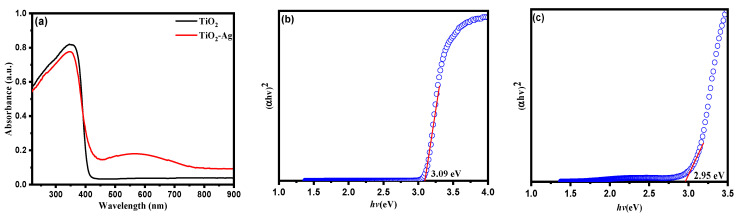
DR UV-vis spectra of (**a**) TiO_2_ and TiO_2_-Ag samples, and the Tauc plot of (**b**) TiO_2_ and (**c**) Ag-modified TiO_2_ nanomaterial.

The diffuse reflectance UV-vis spectrum of the bare TiO_2_ sample showed a broad band at 380 nm (Figure 3a), and the spectrum did not exhibit any specific peaks at higher wavelengths, as it had not absorbed visible light. On the other hand, the spectrum of the Ag-modified TiO_2_ sample showed two absorption peaks at 380 nm and 550 nm, which could have been ascribed to the absorption of the UV and visible light energy, respectively. The absorption edges of TiO_2_ were extended to the visible light region, which indicated that the optical band gap of TiO_2_ decreased after the modification of metallic silver. The optical band gaps of both the bare and Ag-modified TiO_2_ samples were estimated from the Tauc plots by following the relation (Equation (6)) [43].

(6) $${\left(\alpha h\upsilon \right)}^{2}=K\left(h\upsilon -E_{g}\right)\;$$

where *α* is the absorbance value at a particular wavelength (*λ* in nm). *K* = an energy-independent constant, *hν* = incident photon energy (hC/*λ* = 1240/*λ*), *h* = Planck’s constant, C = speed of light, and *E_g_* is optical band gap energy. Equation (6) can be written as Equation (7) for a known function.

(7) $${\left(\alpha \frac{1240}{\lambda }\right)}^{2}=\frac{1240}{\lambda }-E_{g}\;$$

Figure 3b,c was constructed between (*α*1240/*λ*)^2^ and 1240/*λ*; a straight line calculated the *E_g_* extrapolated to the (*α*1240/*λ*)^2^ = 0 axis. The band gap of the Ag-modified TiO_2_ (*E_g_* = 2.95 eV) was inferior to that of bare TiO_2_ (*E_g_* = 3.09 eV), which suggested the enhancement of visible light absorption and the charge carrier efficiency of Ag-modified TiO_2_ in comparison to its parent material [44].

**Figure 4:**
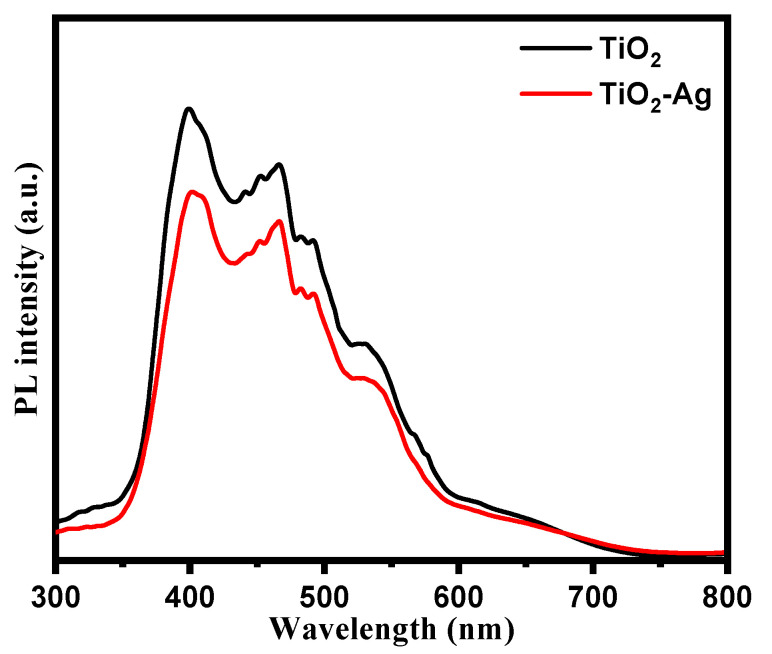
PL spectra of TiO_2_ and Ag-modified TiO_2_-nanomaterial.

Figure 4 shows the PL spectra of bare TiO_2_ and Ag-modified TiO_2_ samples in the 300 to 600 nm range at room temperature. The PL spectra of the Ag-modified TiO_2_ sample exhibited a blue shift in comparison with the bare TiO_2_ sample. This sample showed three promin_ent_ emission peaks at ca. 410 nm, 460 nm, and 480 nm, corresponding to 3.02 eV, 2.69 eV, and 2.58 eV, respectively (Figure 4, red line). The blue emission peak at 410 nm was attributed to the free excitonic emission from the TiO_2_ (band-band photoluminescence phenomenon). The emission peak at 460 nm was due to the oxygen vacancies presented in the Ag-modified TiO_2_, whereas the peak at 480 nm could have been due to the bound excitations of surface defects [45]. In addition, a few minor emission peaks were observed in the range of 450 to 500 nm [46]. The photoluminescence peak intensities were decreased after the modification of TiO_2_ with silver species, indicating the enhanced suppression of e^−^/h^+^ recombination, which was beneficial for hydrogen generation in the photocatalytic processes [47].

**Figure 5:**
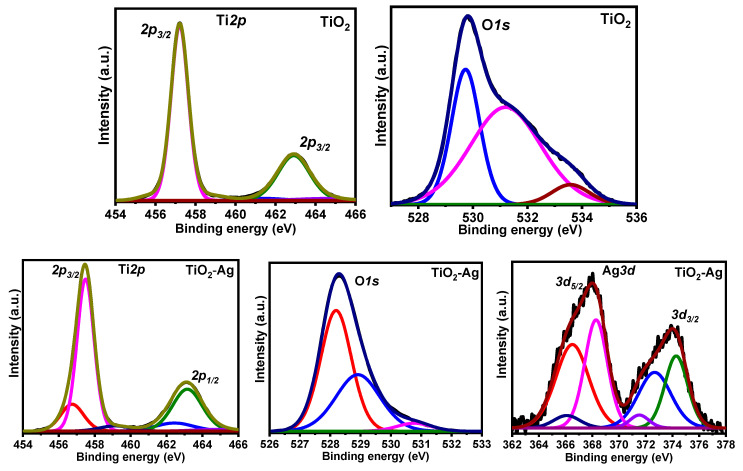
Deconvoluted XPS spectra for TiO_2_ and Ag-modified TiO_2_ samples.

The X-ray photoelectron spectroscopy (XPS) analysis provided an insight into the surface elemental composition of the prepared nanomaterials. Figure 5 depicts the high-resolution deconvoluted O*1s*, Ti*2p*, and Ag*3d* XPS spectra for the synthesized samples. The XPS spectrum of the bare TiO_2_ sample showed the existence of Ti and O elements on the surface. On the other hand, the TiO_2_-Ag sample showed contributions due to the Ti, O, and Ag elements. The presence of doublet characterized the Ti^+4^ oxidation state for TiO_2_ due to Ti 2*p*_3/2_ and Ti 2*p*_1/2_ contributions at 457.1 eV and 462.9 eV, respectively [48]. However, the peak positions for Ti *2p* shifted to higher binding energies (457.5 eV and 463.1 eV) for the TiO_2_-Ag sample due to the decrease in the surface electron density of TiO_2_ after Ag modification. This observation indicated that Ag species interacted with TiO_2_ nanoparticles in this sample. The deconvoluted Ti *2p* XPS spectrum for the TiO_2_-Ag sample yielded two different peaks centered at 458.9 eV and 458.1 eV, ascribed to Ti^4+^ and Ti^3+^ species, respectively. This observation confirmed the existence of strong interaction between Ag and Ti in the TiO_2_-Ag sample. Visible-light-driven TiO_2_-Ag nanostructures may exhibit enhanced photocatalytic activities due to the presence of Ti^3+^ oxides with narrow band gaps. The O*1s* spectra of bare TiO_2_ and TiO_2_-Ag samples are also shown in Figure 5. The deconvoluted O*1s* spectrum for bare TiO_2_ sample showed three peaks at 529.8 eV, 531.2 eV, and 533.5 eV, ascribed to the oxygen of the Ti-O bonds in the TiO_2_, Ti-OH bonds, and surface OH groups, respectively. On the other hand, the O1*s* for the TiO_2_-Ag sample showed a different set of three peaks at binding energies of 528.2 eV, 528.9 eV, and 530.7 eV, corresponding to adsorbed O on Ag, the lattice oxygen of TiO_2_, and the adsorbed OH species, respectively [48].

These observations clearly indicated that the surface structure of the TiO_2_-Ag sample was different from bare TiO_2_. The deconvoluted Ag 3*d* spectrum of the TiO_2_-Ag sample showed three peaks for both Ag 3*d*_5/2_ and Ag 3*d*_3/2_ contributions. The Ag 3*d*_5/2_ peaks appeared at 368.3 eV, 366.5 eV, and 366.0 eV, corresponding to Ag^0^, Ag^+^, and the satellite peak for Ag^0^, respectively [49]. The binding energies of the Ag 3*d* peaks were relatively lower than the bulk Ag species, suggesting a strong interaction between the Ag and TiO_2_. Therefore, there was a clear possibility for the electron transfer from the TiO_2_ nanoparticles to metallic Ag. In addition, diffuse reflectance UV-vis spectra showed a plasmonic absorption band for the Ag^0^ species, indicating that the Ag^0^ species were on the surface of the TiO_2_ particles. The XPS findings showed that abundant Ag nanocrystals developed on the TiO_2_ nanoparticles, and the Ag and TiO_2_ interacted strongly at the interface of the TiO_2_-Ag nanostructures. As a result, the nanostructures were made up of Ti, O, and Ag elements, which agreed with the XRD results.

**Figure 6:**
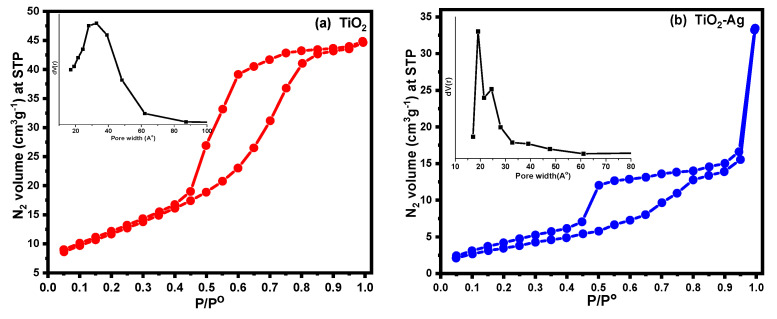
N_2_ adsorption–desorption isotherms for (**a**) TiO_2_ and (**b**) TiO_2_-Ag samples (inset pore size distribution pattern).

It is well known that the porosity of a photocatalyst is one of the physicochemical properties that could influence its photocatalytic efficiency [50]. To investigate the modification of Ag on the textural properties of the prepared TiO_2_ photocatalyst, N_2_ physisorption measurements for bare TiO_2_ and TiO_2_-Ag samples were performed. The N_2_ adsorption–desorption isotherm of the bare TiO_2_ sample exhibited a typical Type-V isotherm with a type D hysteresis loop (Figure 6), indicating the presence of bottleneck mesopores in the sample (IUPAC classification). Interestingly, the TiO_2_-Ag sample showed a unique inverse “S” shape isotherm. The adsorption isotherm showed a convex-shaped increase at its monolayer adsorption in the low-pressure range (P/P^0^ = 0 − 0.05), revealing the presence of micropores, and the multilayer adsorption occurred in the pressure range (P/P^0^ = 0.05 − 0.45). Then, the adsorption increased at the moderate pressure range (P/P^0^ = 0.35 − 0.9); then, at the latter part of the adsorption isotherm, the sharp rising presented a concave shape, indicating that the TiO_2_-Ag sample developed macropores, and capillary condensation occurred in the mesopores, indicating that this sample possessed three pore systems (micro-, meso-, and macropores) [51]. The pore size distribution patterns obtained by the NLDFT method presented in the inset of the adsorption–desorption isotherms, which revealed that the bare TiO_2_ sample possessed unimodal pores with an average pre-width of 2.8 nm. The TiO_2_-Ag sample exhibited a bimodal pore system, with an average pore width of 3 nm. The observed macropores could have been due to the random arrangements of the TiO_2_ and Ag metal nanoparticles creating large-size void spaces. The Brunauer–Emmett–Teller (BET) surface area was determined, and it was observed that the TiO_2_-Ag sample possessed a relatively high surface area (180.2 m^2^/g) compared to bare TiO_2_ (140.9 m^2^/g), probably due to the presence of micropores.

### 3.2. Catalytic Activity of TiO_2_ and Ag-Modified TiO_2_ Nanomaterials for Hydrazine Decomposition

To investigate the catalytic activities of synthesized nanomaterials, hydrazine was chosen as a H_2_ storage material. Initially, hydrazine decomposition (0.05 mol/L) was performed with a fixed amount of catalyst (20 mg) at 298 K under dark and visible light radiation. Then, the H_2_ selectivity (*α*) was calculated according to Equation (8) [52].

(8) $$\alpha =\frac{3\left(n\left(H_{2}+N_{2}\right)/n\left(N_{2}H_{4}\right)\right)}{8}-1\;$$

**Figure 7:**
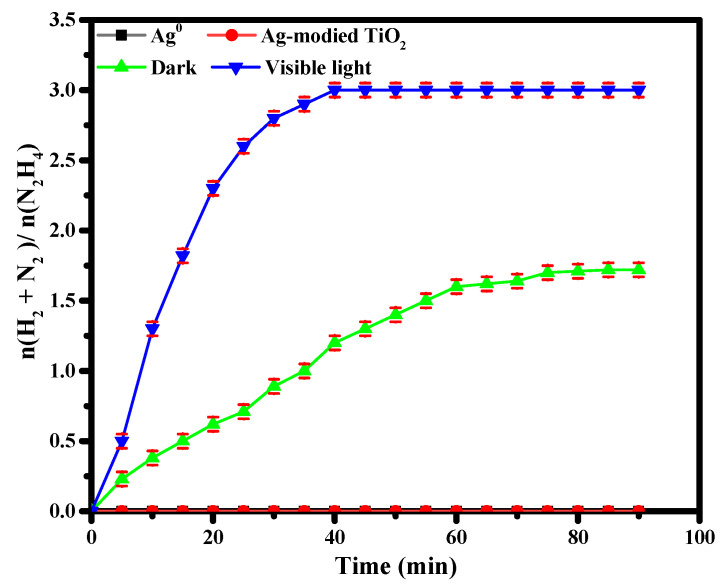
Decomposition of hydrazine with and without light radiation at 295 K.

The observed H_2_ selectivity versus reaction time were plotted in Figure 7. The control experiment (without catalyst) was also carried out for comparison, and no hydrogen evolution was observed, as shown in Figure 7. The progress of the reaction was slow in the dark, and it was completed in 60 min with ca. 50% hydrogen selectivity. Interestingly, no gas formation (H_2_, N_2_, or NH_3_) was observed with bare Ag NPs and bare TiO_2_ samples even after 2 h of reaction under optimized experimental conditions. Nessler’s reagent tests suggested that NH_3_ was not formed, as it is the major side product during reaction. These results were in accordance with the observations of Sing et al., who detailed that the mono-metallic Cu, Fe, Pt, and Pd nanoparticles were not active for hydrazine decomposition in an aqueous medium [53]. The H_2_ selectivity also depended on the molar ratio of metals in the bi-metallic Fe-Pd, Fe-Cu, Co-Cu, Cu-Rh, Cu-Pt, Cu-Ir, and Cu-Pd nanoparticles. In the present study, the H_2_ generation by hydrazine in the dark with an Ag-modified TiO_2_ sample was probably due to the surface plasmon resonance (SPR) mechanism as well as a synergistic effect between the Ag^0^ and TiO_2_ structures [54,55].

**Figure 8:**
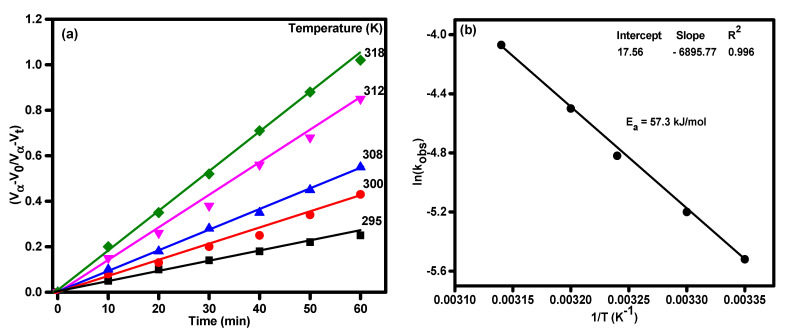
Influence of reaction temperature (**a**) and Arrhenius plot for the H_2_ generation (**b**) in the absence of visible light.

The decomposition of hydrazine was carried out under pseudo-first-order conditions ([hydrazine] ˃ [catalyst]) (Table 1). The decomposition rate constants were calculated by using the well-known first-order rate equation (Equation (5)). The reaction-time profile clearly showed that the decomposition of hydrazine followed first-order kinetics with respect to hydrazine concentration at each temperature under pseudo-first-order conditions. Figure 8a shows the influences of the reaction temperatures (295, 300, 308, 312, and 318 K) on H_2_ generation. The plots of *ln*(*V_α_* − *V*_0_/*V_α_* − *V_t_*) versus time are straight lines and pass through the origin, indicating the first-order dependence with respect to hydrazine concentration at each temperature under pseudo-first-order conditions. The Arrhenius equation (Equation (9)) was employed to estimate the activation energy for hydrazine decomposition over an Ag-modified TiO_2_ catalyst (Figure 8b).

(9) $$\left(lnk_{obs}\right)=-\frac{E_{a}}{8.314\times T}+lnA\;$$

where *k_obs_*, *E_a_*, *T*, and *A* are the rate constant, activation energy, absolute temperature, and Arrhenius factor. The *E_a_* = 57.3 kJ/mol was evaluated from the slope of the Arrhenius plot (Figure 7b). The turnover frequency (*TOF*) was estimated with Equation (10) [56].

(10) $$TOF=\frac{n{\left(N_{2}H_{4}\right)}_{50\%}}{n{\left(Ag-TiO_{2}\right)}_{50\%}\times time_{50\%}}\;$$

The *TOF* values were calculated to be 80, 120, and 190 h^−1^ at 298, 308, and 318 K, respectively. The influence of the pH of the solution was studied (from 9.5, 10.0, and 11.5) at a fixed hydrazine concentration (0.5 mol), catalyst (20 mg), and temperature (298 K) in the dark. It was experimentally tested that the pH of the Ag-modified TiO_2_ and hydrazine solution was moderately acidic (pH ca. 5.4) and alkaline (pH ca. 8.4) in nature. The pH was adjusted by adding standard NaOH solution, which has been used as a promoter for the decomposition of hydrazine. The pH had a significant role in the catalytic dehydrogenation reaction (Table 1).

Normally, TiO_2_ is an amphoteric metal oxide; however, the hydroxylated surface of TiO_2_ tends to donate protons by dissociating water, binding the HO^−^ ions and releasing H^+^ ions. The increased H^+^ ions in the solution resulted in a decrease in pH. It is known that hydrazine is a weak Brønsted base (pK_b_ = 8.0) and mainly exists in the protonated form at pH ca. 7.0. The acid–base equilibrium in an aqueous suspension of Ag-modified TiO_2_ and hydrazine was due to the acidic functional groups presented on the surface of TiO_2_ (Equations (11) and (12)).

(11) $$TiO_{2}+H_{2}O↔TiO_{2}-OH+H^{+}\;$$

(12) $$NH_{2}-NH_{3}^{+}↔NH_{2}-NH_{2}+H^{+}\;$$

**Figure 9:**
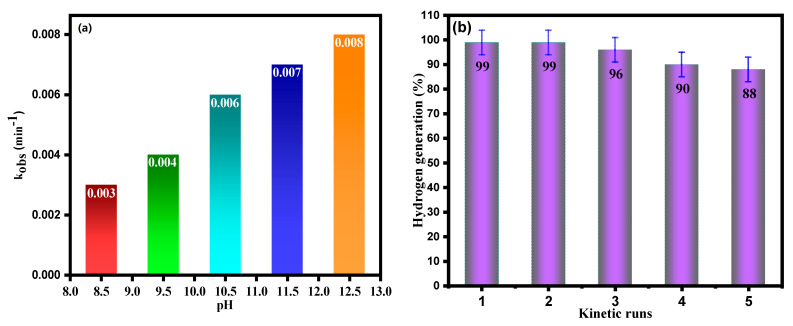
Effects of pH (**a**,**b**) reusability of catalyst for H_2_ generation.

As the HO^−^ ions’ concentration increased, the catalyst’s surface and hydrazine became active due to the involvement of various surface reactions. The percentage of protonated hydrazine decreased with a pH increase, and equilibrium shifted towards the right at a higher pH (Equation (10)). As a result, H_2_ generation rates increased with an increase in the pH of the aqueous hydrazine solution (Figure 9a) [33].

The rate-determining step for hydrazine decomposition [NH_2_-NH_2_ → NH_2_-NH^•^ + H^•^] was accelerated at high pH due to the inhibition of NH_3_ formation. The reusability of the Ag-modified TiO_2_ was estimated under the optimized experimental conditions. After the completion of the first cycle of the activity test, the catalyst was recovered, washed with water, dried at 90 °C for 2 h, and reused for the next cycle. The catalyst was reused for five consecutive kinetic experiments with the same hydrazine concentration. The results of the reusability tests are presented in Figure 9b. It was observed that the catalytic activity remained unchanged for all cycles, which indicated that the Ag-modified TiO_2_ was a stable catalyst with excellent catalytic performance. However, the activity was slightly decreased after five recycle kinetic runs, and the decrease in activity could have been due to two reasons; (i) loss of catalyst amount during the recycling experiments and/or (ii) oxidative conversion of Ag^0^ under alkaline reaction media, as Ag^0^ nanoparticles are not stable in an alkaline reaction condition [57].

**Scheme 1:**
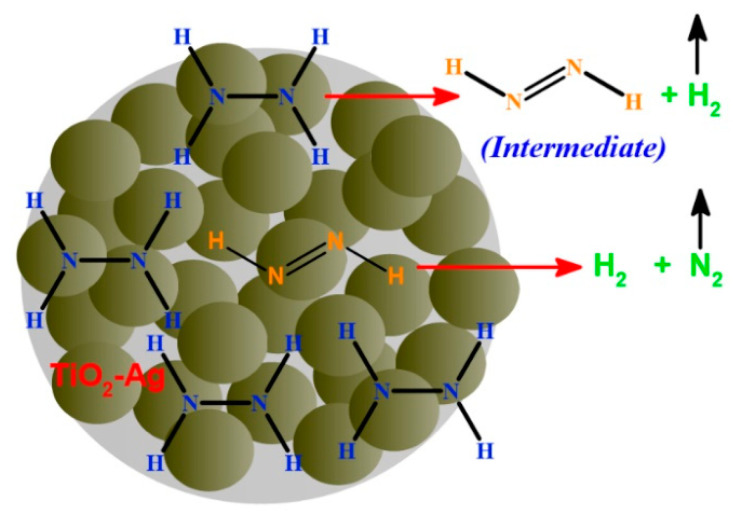
Schematic representation of thermal decomposition of hydrazine over Ag-TiO_2_ composite.

Scheme 1 represents the possible mechanism for the thermal decomposition of hydrazine over the surfaces of the Ag-TiO_2_ particles. Initially, the hydrazine molecules adsorbed onto the surface of Ag/TiO_2_ particles. Then, the adsorbed hydrazine molecules underwent N-N and N-H bond cleavages [8], which led to the formations of final decomposition products of hydrazine, i.e., H_2_ and N_2_ gases. Hydroxyl functional groups on the TiO_2_ surface could contribute to the formation and fixation of Ag^0^ NPs but also enhance the synergy between Ag metal NPs and TiO_2_, which could yield high catalytic activity with 100% H_2_ selectivity.

**Figure 10:**
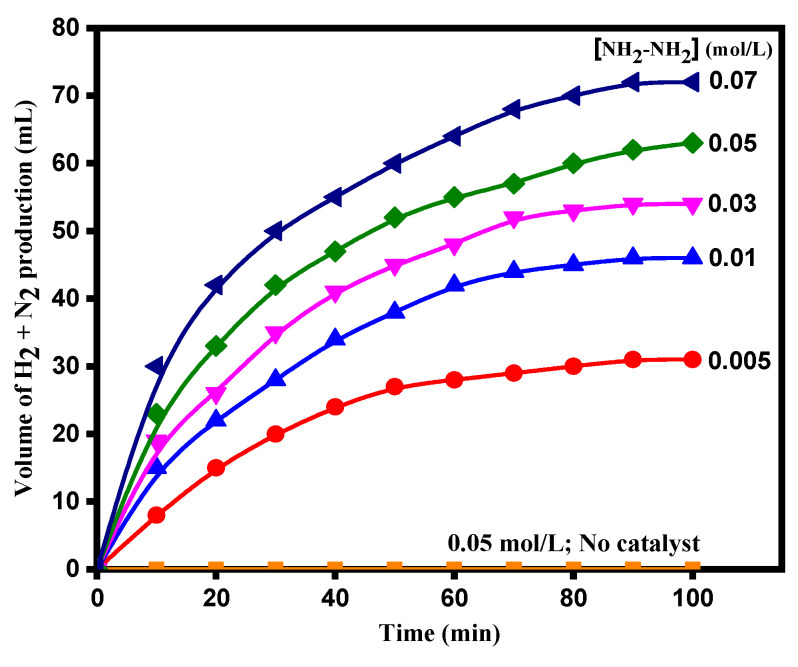
Volume of generated gas versus time plots for the Ag/TiO_2_-assisted degradation of hydrazine in presence of visible light.

Additional experiments were performed to study the effect of hydrazine concentration on the decomposition ability of the TiO_2_-Ag catalyst. Figure 10 shows the effect of hydrazine concentration on the TiO_2_-Ag-assisted photocatalytic degradation of hydrazine into H_2_ and N_2_. The volume of generated gases mixture increased with increasing hydrazine concentration. The values of rate constants were calculated by using the pseudo-first-order kinetic rate law (Equation (5)) to establish the kinetics of hydrazine decomposition on the surface of the TiO_2_-Ag catalyst. The values of the rate constants as a function of hydrazine concentration, amount of catalyst, temperature, and pH are given in Table 1. The hydrazine decomposition followed first-order kinetics with respect to hydrazine concentration. Therefore, the complete decomposition of hydrazine occurred, and there was no possibility for a side reaction (oxygen reduction by hydrazine). Thermodynamically, the decomposition of hydrazine by oxygen to H_2_O and N_2_ was possible (N_2_H_4_ + O_2_ → 2H_2_O + N_2_), but the same reaction was very slow in an aqueous system (hydrazine + O_2_ + H_2_O) [58], which might have been due to the protonation of hydrazine in water (Equation (12)). The escaping tendency of N-H bond decreased in the protonated hydrazine due to the presence of a positive charge on the nitrogen. Thus, a suitable catalyst was required for the complete dehydrogenation of hydrazine into N_2_ and H_2_ [32,53].

At this juncture, it is important to study the roles of reaction conditions on selective hydrazine decomposition, as it is well known that the presence of molecular oxygen in the reaction system results in the reduction of oxygen by hydrazine to produce N_2_ and H_2_O [58]. Therefore, some kinetic experiments were performed to determine the stability of hydrazine in presence of water and oxygen. The results of these experiments are summarized in Table 2. There was no hydrazine decomposition activity when the water, visible light, and molecular oxygen were presented in the reaction mixture. Some experiments were repeated under anaerobic conditions. For this purpose, pure helium gas was bubbled through the reaction mixture (containing TiO_2_-Ag catalyst and water) for 30 min to remove the oxygen. The 0.05 mol/L hydrazine was added to the reaction vessel, and the evolution of N_2_/H_2_ was monitored through the water displacement method at different time intervals. No gases were generated even after 1 h under anaerobic conditions. Therefore, it was clear that the hydrazine was stable in water, and oxygen was not reduced by the hydrazine with and without the TiO_2_-Ag catalyst. The observed results were in good agreement with the results reported by Gaunt and Wetton, with respect to the reaction of hydrazine and oxygen in water [58]. These authors reported that the direct reaction between hydrazine and oxygen at 298 K was very slow (8% in 90 h), and the slow reaction may have been catalyzed by different catalysts. Moreover, the dehydrogenation of hydrogen was strongly dependent on the nature of the catalyst, as well as other reaction conditions [32,55,58]. In order to establish the role of molecular O_2_, some experiments were also performed with dissolved O_2_ in an aqueous solution under various experimental conditions. We did not observe the formation of H_2_ and N_2_ gases in the mixture for ca. 1 h (Table 2).

**Figure 11:**
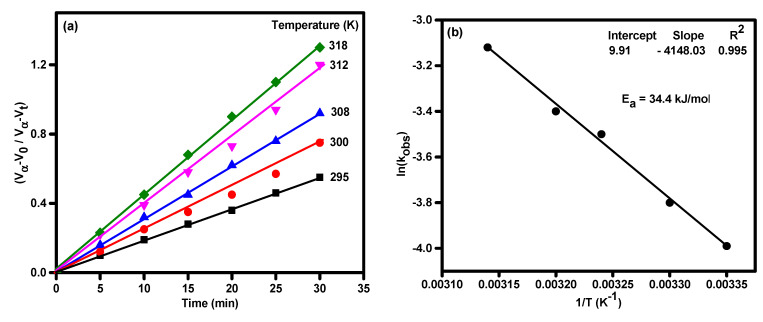
Effects of temperature (**a**) and Arrhenius plot for the H_2_ generation (**b**) under visible light radiation.

The H_2_ production rates were accelerated with increased reaction temperature under visible light radiation (Figure 11a). The activation energy (*E_a_*) was calculated from the slope of the Arrhenius plot (Figure 11b) observed as 34.4 kJ/mol. These results indicated that the Ag-modified TiO_2_ catalyst offered high photocatalytic H_2_ generation from hydrazine in visible light radiation. Figure 8 clearly shows that the catalytic hydrazine decomposition also occurred in the dark. Therefore, hydrazine decomposition process did not require photo energy to initiate [29,59]. The effect of hydrazine concentration was studied, ranging from 1.0 × 10^−2^ to 7.0 × 10^−2^ mol/L, with a catalyst amount of 20 mg with pH 9.5 at 295 K. Table 1 shows the rates of hydrazine decomposition, and the reaction followed the first-order kinetics with hydrazine concentration. Oliaee et al. also reported that the decomposition rate (generation of N_2_ + H_2_) depended on the initial concentration of hydrazine [60].

### 3.3. Mechanism of Photochemical Hydrazine Decomposition

**Figure 12:**
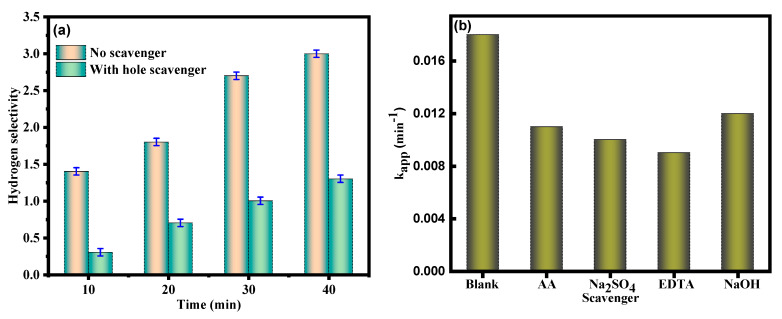
(**a**) Photocatalytic H_2_ generation from hydrazine by using Ag-modified TiO_2_ with EDTA hole scavenger, (**b**) Different scavengers used during photocatalytic hydrazine decomposition over TiO_2_-Ag catalyst. Na_2_SO_4_.

For plasmonic metal nanoparticles, the color and charge separations on their surface were due to the collective oscillations of free electrons derived from the localized surface plasmon resonance (SPR) effect, and the surface charge density was partially localized. Therefore, the catalytic activity of Ag-modified TiO_2_ might have been due to the combination of synergistic and SPR effects. During the photocatalysis process, target molecules react with photogenerated holes at the valence band (VB) of the semiconductor [61]. To establish the role of photogenerated holes of TiO_2_ in the degradation of hydrazine, EDTA, a hole scavenger, was used [62] during the photocatalytic process. The H_2_ selectivity was estimated in presence of EDTA (0.5 mol/L) under similar experimental conditions, and the obtained results are presented in Figure 12.

Figure 12a shows that H_2_ generation decreased when the hole scavenger was presented in the reaction mixture, which clearly indicated the role of photogenerated holes in hydrazine decomposition. Furthermore, different scavengers, such as ascorbic acid, sodium sulfite, and sodium hydroxide (0.5 mol/L), were also tested under visible light radiation to determine the role of oxidation sites for photo-assisted hydrazine decomposition [63]. Figure 12b demonstrates the decreases in *k_app_* values due to presence of scavengers. Thus, the reactive holes of TiO_2_ are the most effective sites for the decomposition of hydrazine, indicating that the degradation of hydrous hydrazine progressed by the holes generated by the visible light radiation at the *VB* of TiO_2_. The *CB* potential (*E_CB_*) and *VB* potential (*E_VB_*) of Ag-doped TiO_2_ were determined by applying Equations (13) and (14) [64].

(13) $$E_{CB}\left(TiO_{2}\right)=EN\left(TiO_{2}\right)-EX-0.5E_{g}\left(TiO_{2}\right)\;$$

(14) $$E_{VB}\left(TiO_{2}\right)=E_{g}\left(TiO_{2}\right)-E_{CB}\left(TiO_{2}\right)\;$$

where *E_CB_* = potential of conduction band, *E_VB_* = potential of valence band, *EN* = electronegativity of TiO_2_ (5.81), and *E_g_* = band gap energy (2.09 eV) of TiO_2_. *E_X_* is the energy of free electrons on the hydrogen electrode scale (ca. 4.5 eV). The values of *E_CB_* and *E_VB_* were calculated and found to be −0.16 eV and 3.1 eV, respectively. The higher value of *E_VB_* could result in the enhanced oxidizing power of the photocatalyst toward hydrazine under visible light exposure. The (*E_CB_* value = −0.16 eV was more negative than the H^+^/H_2_ reduction potential, indicating that the photogenerated electrons on the conduction band of TiO_2_ due to the SPR effect of Ag^0^ could effectively reduce H^+^ to H_2_. It is well known that the 0.00 V and −0.41 V reduction potentials are needed for H_2_ production (H^+^/H_2_) at pH 0.0 and in an aqueous solution at pH 7.0, respectively [63]. The negative and positive values of *E_CB_* and *E_VB_* were quite enough to promote reduction and oxidation steps to generate the required electrons and protons [65].

**Scheme 2:**
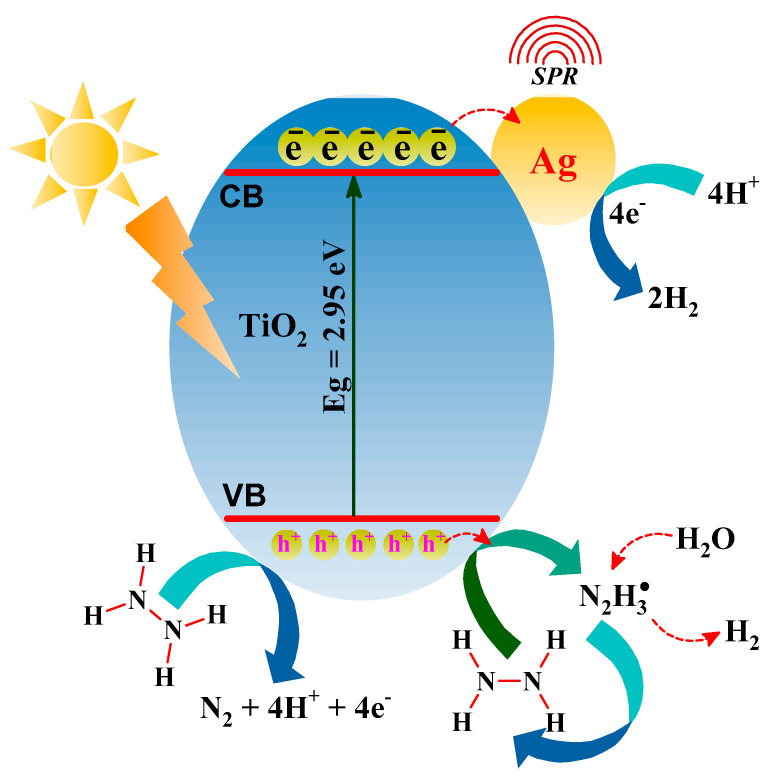
The hydrazine photocatalytic decomposition under visible light radiation over Ag-modified TiO_2_.

Unmodified TiO_2_ and Ag^0^ individually could not be able to catalyze the hydrazine decomposition due to the wide band gap and lack of photogenerated holes under tested reaction conditions. The modified TiO_2_ with Ag^0^ (TiO_2_-Ag) was active and produced H_2_ due to hydrazine decomposition, but decomposition rates were low in the absence of light. The TiO_2_-Ag semiconductor absorbed visible light radiation and produced e^−^/h^+^ pairs; the photogenerated e^−^ transfer occurred from VB to the CB of TiO_2_. Thus, Ag^0^ nanoparticles acted as electron traps, which were essential for efficient H_2_ generation. It was possible to propose a plausible reaction mechanism for photocatalytic decomposition of hydrazine over Ag-modified TiO_2_ based on the observed results from the activity and characterization studies (Scheme 2). The N-H and N-N bonds of hydrazine are attached to the surface of TiO_2_ due to the adsorption of hydrazine. The surface heterogeneity activated the H-N bonds of hydrazine; then, various intermediates (NH_2_-NH^•^, HN^•^-NH^•^, HN=NH, and ^•^N-NH) were formed due to the reactivity of water molecules, which was the key step to hydrazine decomposition in the presence of light [29]. The Schottky barrier could easily form between the Ag^0^ and TiO_2_‘s crystal structure due to the high Fermi level of TiO_2_ compared to metallic silver particles. However, the photogenerated e^−^ overcame the Schottky barrier and transferred through the Ag^0^-TiO_2_ interface to the CB of TiO_2_. The e^−^ transfer was much faster in the plasmonic nanomaterials due to the SPR effect [66]. A comparison of the catalytic activity of the Ag-modified TiO_2_ catalyst with the other reported catalysts is shown in Table 3. The activation energy was lower in case of the TiO_2_-Ag than other reported catalysts, indicating that the Ag^0^ plasmonic nanoparticles enhanced the photocatalytic activity of bare TiO_2_.

## 4. Conclusions

In conclusion, an Ag-modified TiO_2_ nanomaterial was successfully prepared using a simple, one-pot synthesis method. The synthesized nanomaterial was used as a novel catalyst for H_2_ production via the thermal and photocatalytic decomposition of an aqueous hydrazine solution under visible light for the first time. The Ag modification decreased the band gap energy of TiO_2_, enhancing the absorption of UV and visible light and boosting the photocatalytic efficiency. The modified TiO_2_ with plasmonic Ag^0^ exhibited excellent catalytic and photocatalytic activities for H_2_ production compared to bare TiO_2_ and Ag^0^ catalysts. TiO_2_ modified with Ag NPs was found to enhance the catalytic performance of TiO_2_ and shift the photoresponse of TiO_2_ from the UV light to the visible light region. In addition, Ag NPs played an important role in reducing the charge recombination of TiO_2_. The Ag NPs also helped in trapping the excited electrons at the surface of the photocatalyst, acted as an electron acceptor, and played a significant role in electron-hole separation. Furthermore, this study provided new insight into the formation of noble-metal-modified semiconductor materials with enhanced charge separations and resulted in speeding up H_2_ production rates.

## Figures and Tables

**Table 1:** Values of rate constants for H_2_ generation from hydrazine by Ag-modified TiO_2_ with and without visible light.

Without Visible Light Radiation					
Hydrazine (mol/L)	Catalyst (g)	pH	Temperature (K)	*k_obs_* (min^−1^)	R^2^
1.0 × 10^−2^	0.0	9.5	295	No degradation	
1.0 × 10^−2^	0.02	9.5	295	0.002	0.996
3.0 × 10^−2^	0.02	9.5	295	0.004	0.993
5.0 × 10^−2^	0.02	9.5	295	0.007	0.997
7.0 × 10^−2^	0.02	9.5	295	0.009	0.991
3.0 × 10^−2^	0.03	9.5	295	0.005	0.995
3.0 × 10^−2^	0.04	9.5	295	0.008	0.992
3.0 × 10^−2^	0.04	8.5	295	0.003	0.992
3.0 × 10^−2^	0.02	10.5	295	0.006	0.992
3.0 × 10^−2^	0.02	11.5	295	0.007	0.991
3.0 × 10^−2^	0.04	12.5	295	0.008	0.992
3.0 × 10^−2^	0.02	9.5	300	0.006	0.996
3.0 × 10^−2^	0.02	9.5	308	0.007	0.994
3.0 × 10^−2^	0.02	9.5	312	0.009	0.994
3.0 × 10^−2^	0.02	9.5	318	0.017	0.998
**Under Visible Light Radiation**					
5.0 × 10^−3^	0.02	9.5	295	0.009	0.995
1.0 × 10^−2^	0.02	9.5	295	0.012	0.992
3.0 × 10^−2^	0.02	9.5	295	0.018	0.998
5.0 × 10^−2^	0.02	9.5	295	0.025	0.996
7.0 × 10^−2^	0.02	9.5	295	0.042	0.991
3.0 × 10^−2^	0.03	9.5	295	0.032	0.993
3.0 × 10^−2^	0.03	8.5	295	0.027	0.993
3.0 × 10^−2^	0.04	9.5	295	0.062	0.995
3.0 × 10^−2^	0.02	10.5	295	0.083	0.994
3.0 × 10^−2^	0.02	11.5	295	0.093	0.991
3.0 × 10^−2^	0.03	12.5	295	0.098	0.993
3.0 × 10^−2^	0.02	9.5	300	0.021	0.998
3.0 × 10^−2^	0.02	9.5	309	0.030	0.999
3.0 × 10^−2^	0.02	9.5	312	0.039	0.998
3.0 × 10^−2^	0.02	9.5	318	0.044	0.999

**Table 2:** Effects of water, oxygen, visible light, and TiO_2_-Ag on the dehydrogenation of hydrazine at 295 K in alkaline solution.

Reaction Conditions	pH	Observations
NH_2_-NH_2_ + water	9.5	No production of H_2_/N_2_, time = 1 h
NH_2_-NH_2_ + water + O_2_	9.5	No production of H_2_/N_2_, time = 1 h
NH_2_-NH_2_ + water	9.5	No production of H_2_/N_2_, time = 1 h
NH_2_-NH_2_ + water + visible light	9.5	No production of H_2_/N_2_, time = 1 h
NH_2_-NH_2_ + water + O_2_ + TiO_2_	9.5	No production of H_2_/N_2_, time = 1 h
NH_2_-NH_2_ + water + O_2_ + TiO_2_-Ag	9.5	No production of H_2_/N_2_, time = 1 h
NH_2_-NH_2_ + water + O_2_ + TiO_2_-Ag + visible light	9.5	No production of H_2_/N_2_, time = 1 h
NH_2_-NH_2_ + water + TiO_2_-Ag	9.5	45% H_2_/N_2_ production, time = 1 h, *k_obs_* = 0.007 min^−1^
NH_2_-NH_2_ + water + TiO_2_-Ag + visible light	9.5	100% H_2_/N_2_ production, time = 40 min, *k_obs_* = 0.025 min^−1^

**Table 3:** The catalytic activity of different catalysts for the decomposition of aqueous hydrazine.

Catalyst	Activation Energy at Different Conditions	Reference
Ag^0^	No decomposition	Present work
TiO_2_	No decomposition	Present work
Ag-doped TiO_2_	*E_a_* = 57.3 kJ/mol in the dark	Present work
Ag-doped TiO_2_	*E_a_* = 34.4 kJ/mol under visible light	Present work
In_4_Ni	*E_a_* = 38.9 kJ/mol in the dark	[67]
Ni_0.8_Pt_0.2_/DT-Ti_3_C_2_Tx	*E_a_* = 64.3 kJ/mol in the dark	[68]

## Data Availability

Not applicable.
